# Factors Associated with Results and Conclusions of Trials of Thiazolidinediones

**DOI:** 10.1371/journal.pone.0005826

**Published:** 2009-06-08

**Authors:** Gail Rattinger, Lisa Bero

**Affiliations:** 1 Department of Pharmaceutical Health Services Research, School of Pharmacy, University of Maryland at Baltimore, Baltimore, Maryland, United States of America; 2 Clinical Pharmacy and Health Policy, University of California San Francisco, San Francisco, California, United States of America; University Paris 7, France

## Abstract

**Background:**

When a sponsor funds a study of two competing drugs in a head-to-head comparison, the results and conclusions are likely to favor the sponsor’s drug. Thiazolidinediones, oral medications used for the treatment of type 2 diabetes, are one of the most costly choices of oral anti-diabetic medications, yet they do not demonstrate clinically relevant differences in achieving lower glycosylated hemoglobin levels compared to other oral antidiabetic drugs. Our aim is to examine associations between research funding source, study design characteristics aimed at reducing bias, and other factors with the results and conclusions of randomized controlled trials (RCTs) of thiazolidinediones compared to other oral hypoglycemic agents.

**Methods and Findings:**

This is a cross-sectional study of 61 published RCTs comparing a thiazolidinedione (glitazone) to another anti-diabetic drug or placebo for treatment of type 2 diabetes. Data on study design characteristics, funding source, author’s financial ties, results for primary outcomes, and author conclusions were extracted. Univariate logistic regression identified associations between independent variables and results and conclusions that favored the glitazone. Of the RCTs, 59% (36/61) were funded by industry, 39% (24/61) did not disclose any funding. Common study design weaknesses included inadequate blinding and lack of concealment of allocation. Trials that reported favorable glycemic control results for the glitazone were more likely to have adequate blinding (OR (95% CI)  = 5.42 (1.46, 21.19), p = 0.008) and have a corresponding author with financial ties to the glitazone manufacturer (OR (95% CI)  = 4.12 (1.05, 19.53); p = 0.04). Trials with conclusions favoring the glitazone were less likely to be funded by a comparator drug company than a glitazone company (OR (95% CI)  = 0.026 (0, 0.40), p = 0.003) and less likely to be published in journals with higher impact factors (OR (95% CI)  = 0.79 (0.62, 0.97), p = 0.011). One limitation of our study is that we categorized studies as funded by industry based on each article’s disclosure which could underestimate the number of industry sponsored studies and personal ties of investigators. Additionally, our study did not include any head-to-head comparisons of one glitazone to another.

**Conclusions:**

Published RCT comparisons of glitazones with other anti-diabetic drugs or placebo are predominantly industry supported and this support, as well as the financial ties of study authors, appears to be associated with favorable findings.

## Introduction

Randomized controlled trials are often used to compare the efficacy of two drugs. However, factors such as trial design and subsequent execution, data analysis, and data presentation can introduce bias and discrepancies in reported outcomes [1], [2]. In addition, publication bias occurs through publication of either partial results or statistically significant information only, and is difficult to measure [3], [4].

Pharmaceutical company sponsored trials comparing the sponsor’s drug to a placebo are more likely to report favorable conclusions for the sponsor’s drug [5]–[8]. However, many previous studies of bias have focused on single study design features and not on the aggregated causes of bias that result in favorable results and conclusions [9]–[11]. For example, many studies of association of funding source and favorable outcomes have not explored the effect of comparator drug selection and dosing in head -to -head drug comparisons. Bias introduced by inadequate randomization, concealment of allocation, blinding, or comparator drug choice and dosing, for example, could also be associated with publication of statistically significant results [8], [12]. Heres et al. [2] and Bero et al.[13] have shown that when a sponsor funds a study of two competing drugs in a head-to-head comparison, the results and conclusions are more likely to favor the sponsor’s drug.

Thiazolidinediones, oral medications used for the treatment of type 2 diabetes, provide an interesting class of drugs to examine sources of bias in drug comparison trials. Type 2 diabetes incidence and prevalence is rapidly increasing globally and when uncontrolled can result in many costly macro- and microvascular comorbidities [14], [15]. Thiazolidinediones have been cited as one of the most costly choices of oral anti-diabetic medications, providing a lower magnitude of glycemic control than several other therapeutic drug classes used for type 2 diabetes [14], [16], [17]. Recent systematic reviews found that these agents did not demonstrate clinically relevant differences in their ability to lower glycosylated hemoglobin levels compared to other oral antidiabetic drugs [18], [19]. Thiazolidinediones also have the potential to affect lipid profiles, a common metabolic co-morbidity with diabetes [20]–[22]. Specifically, pioglitazone can improve, while rosiglitazone can negatively affect lipid values. Although glitazones are often used as combination therapy when glycemic control has not been achieved with at least one other oral agent, they are also approved for monotherapy [17], [20]–[22]. An alternative choice of third agent for glycemic control is often insulin, however; many patients prefer oral agents and to avoid initiating insulin therapy as long as possible [17], [22]. We chose to focus on thiazolidinedione trials for two reasons: 1. There are oral agents from the biguanide and sulfonylurea classes that provide equivalent glycemic control and are available as generic drugs [14], [16], [18], [19]and 2. Most insulin therapy offers a less costly alternative to the addition of a thiazolidinedione as a third oral agent to provide glycemic control [14], [17], [22].

This cross-sectional study examines the results and conclusions published in randomized controlled trials (RCTs) of glitazone- placebo comparisons and glitazone-alternative anti-diabetic drug comparisons, and their associations with trial funding source, study design features aimed at reducing bias, and other factors. We hypothesized that results and conclusions of trials are more likely to favor the study sponsor’s drug, and that design features such as randomization, blinding, and analysis technique, as well as author’s financial ties, are associated with significant results favoring the study sponsor’s drug.

## Methods

### Search strategy

We searched PubMed and the Cochrane Library to identify randomized controlled trials published between January 1996 and December 2006 using the MeSH terms and drug names “pioglitazone and diabetes” and “rosiglitazone and diabetes.” with limits of ‘clinical trial, ‘humans’, ‘all adult: 19+ years’ and ‘English’. The search was limited to these dates because the first published thiazolidinedione trials [23] of troglitazone, which was subsequently removed from the market in 2000 and consequently is not included in our study, started appearing around 1996. The first trials on rosiglitazone and pioglitazone were published in 1999. Reference lists of all potentially relevant articles identified through the PubMed search and a recent meta-analysis [24] and systematic review [18] were also reviewed.

### Inclusion and Exclusion Criteria

All abstracts were reviewed and studies were selected for inclusion based on the following criteria: 1) type 2 diabetes mellitus diagnosis; 2) adult study population >18 years of age; 3) glycemic control and/or lipid level measures as outcomes; 4) original trial data were presented; 5) pioglitazone or rosiglitazone were compared to either placebo or another anti-diabetic drug or combination from a different drug class; and 6) one study arm did not contain a glitazone. Because we were interested in looking at clinical trials examining the efficacy of pioglitazone or rosiglitazone as compared to other therapeutic classes of antidiabetic drugs or placebo, we also excluded head-to-head studies of the glitazones by requiring that inclusion criteria 5 and 6 were met.

Articles were excluded based on the following criteria: 1) diagnoses other than type 2 diabetes mellitus including a pre-diabetes diagnosis such as impaired glucose tolerance, polycystic ovary syndrome, HIV lipodystrophy, prior gestational diabetes without current DM diagnosis, 2) pharmacokinetics or pharmacodynamics studies; 3) studies that did not include original trial data such as comments and letters to the editor, reviews, study design and rationale only, re-analyses of previous trial data; 3) trials without primary outcomes focused on improved glycemic control measures and/or lipid level measures such as reduced carotid intima media thickness; 4) trials with troglitazone, as it has been removed from the market due to hepatic toxicity and is not relevant to current drug therapy selection; 5) trials in which dosing ranges were compared without a placebo arm; 6) comparisons of either pioglitazone or rosiglitazone to non-drug interventions such as diet, exercise, improved memory and increased energy level; 7) studies in which a glitazone was present in all study arms; 8) trials of less than 12 week duration as shorter durations do not allow accurate assessment of changes in glycosylated hemoglobin (HgbA1C) value, a key measure of changes in glycemic control and 9) *in-vitro* and mechanistic studies. Discrepancies about inclusion were discussed by both authors until consensus was reached. A chance of bias in selection is that neither coder was blinded to study results or conclusions.

Although no exact duplicate publications were found, we identified 22 publications that either re-analyzed data from a combination of trials, reported on different outcomes from the same trial or were sub-analyses of data sets. The publication with the earliest publication date was used, unless a later publication included more data of interest; for example inclusion of both glycemic control and lipid level measures in a single publication.

### Data extraction

The first author (G.B.R.) extracted all data from each article. All of the coding was discussed with a second coder (L.A.B.) and results were adjudicated to reach agreement. As before, there is some chance that bias was introduced as neither coder was blinded to study results or conclusions.

We extracted data on the following publication characteristics shown to be independently related to favorable results or conclusions of some drug studies [2], [5], [7], [10], [25], [26]. The association of these characteristics with the results and conclusions of thiazolidinedione trials is uncertain.

### Journal characteristics

#### Peer Review Status

Every article was classified as peer reviewed, non- peer reviewed or unknown by using information provided on the journal’s website regarding the publication review process. Articles were classified as peer reviewed if the website stated that the journal had a peer review process or that submitted manuscripts were evaluated by at least one expert in the field; otherwise the publication was classified as non-peer reviewed. If no information could be found on a particular journal, we classified peer review status as unknown.

#### Impact Factor

Impact factors for journals were obtained from the Institute for Scientific Information, 2005 data [27].

### Study characteristics

#### Comparison Groups

Each article was classified as either a drug vs. placebo-control or drug-drug comparison trial. Drug-drug comparison trials which explicitly stated that they were non-inferiority designs were noted. The “test” drug was defined as the glitazone in every trial.

#### Sample Size

We recorded the number of patients included in the analysis of each trial.

### Study design characteristics aimed at reducing bias

We evaluated the following study design attributes aimed at reducing bias [28]:

#### Randomization and concealment of allocation

We classified each trial as having adequate (score of 1) or inadequate (score of 0) randomization and concealment of allocation based on the description of the method of randomization. Specifically, the mere mention of randomization in the methods section was insufficient to receive an adequate randomization score. A detailed description of the way concealment of allocation and randomization were achieved was necessary to receive an adequate score. For example, if the methods described central randomization by a third party or the use of sealed, opaque envelopes, the trial received a score of adequate. If there was no description or mention of randomization, the paper received an inadequate score.

#### Blinding

We classified each trial as having adequate (score of 1) or inadequate (score of 0) blinding of subjects, study investigators, study personnel administering therapy or assessing trial outcomes, and statisticians. A description of blinding had to be given to receive an adequate score; otherwise this attribute was coded as inadequate.

#### Intention-to-treat analysis

We classified each trial as having adequate (score of 1) or inadequate (score of 0) intention-to-treat analysis. If the paper stated that it used an intention-to–treat analysis , it received an adequate score as it was often unclear whether or not exclusions actually occurred. Thus, we likely overestimated the occurrence of true intention-to-treat analysis.

#### Follow-up

We classified each trial as having adequate (score of 1) or inadequate (score of 0) follow-up. Follow-up of ≥75% was required to receive an adequate score.

#### Appropriate dosing range

We classified each trial as having adequate or inadequate drug dosages using standard dosing ranges [17], [22].

### Outcome Measures

#### Coding of Study Results

We examined glycemic control and lipid profile outcomes measures. For glycemic control results we extracted data on glycosylated hemoglobin (HgbA1C) percentages and fasting (FPG/FBG) and/or post-prandial plasma or blood (PPG/PBG) glucose levels. For lipid level results we used total cholesterol (TC), low density cholesterol (LDL-c), high density cholesterol (HDL-c), and triglyceride (TG) levels.

For each article, results reported for each outcome of interest were categorized as 1) favorable to the “test” drug (glitazone) if it was statistically significant (p<0.05 or confidence intervals that excluded no difference) and in the direction of the glitazone being more efficacious, 2) no difference / about equal if it was statistically insignificant, or 3) unfavorable if the result was statistically significant in the direction of the placebo or comparator drug being more efficacious. We coded results from non-inferiority studies as favorable when results were equivalent or better between the glitazone and its comparison drug. Results for primary outcomes were coded separately for glycemic control results and lipid level results. These were then classified independently as favorable for glycemic control if the overall glycemic control results favored the glitazone and as favorable for lipid level profile if the overall lipid level results favored the glitazone. For analysis, we categorized results as favorable or not, combining the about equal/ no difference and unfavorable categories.

#### Coding of Study Conclusions

Conclusions, reported in the discussion section in published papers, were abstracted and coded. Conclusions were classified as 1) favorable, if the glitazone therapy was preferred over the placebo or comparator drug therapy, 2) no difference / about equal, if there was no preference stated for the glitazone drug therapy over the placebo or comparator drug therapies or 3) unfavorable, if the placebo or comparator drug therapies were preferred over the glitazone therapy. Non-inferiority studies were coded as favorable if the glitazone therapy was deemed equivalent to or better than the comparator therapies, as the goal of these trials is to show that drug therapies are equivalent and we are not assessing the size of the effect. When an article did not clearly state a preference for one therapy over another it was coded as no difference / about equal. For analysis, we categorized conclusions as favorable or not, combining the about equal/ no difference and unfavorable categories.

### Author characteristics

#### Institutional Affiliation

Corresponding author affiliation was obtained from the article and classified as 1) “test” drug company (glitazone company), 2) any other industry, 3) academic or hospital or 4) unknown.

### Funding information

#### Funding source

Funding source(s) of published studies were categorized as 1) “test” drug (glitazone), 2) any other industry funding, 3) drug company plus other funding source(s) (mixed funding sources), 4) all other funding (no drug company funding), or 5) no funding disclosure. All categories that applied were coded, so studies that received funding from a glitazone manufacturer as well as other industry funding could be differentiated from studies that received funding from a single drug company. Due to our sample size, studies that received any funding from the “test” drug company (N = 4) were ultimately combined with those funded exclusively by the “test” drug company (N = 26).

#### Financial Ties

We extracted and coded data about the disclosed financial ties of the first and corresponding author as 1) any financial ties disclosed with the sponsor of the study, 2) any financial ties disclosed with any other drug company, 3) “test” drug (glitazone) company employee, 4) any other drug company employee and 5) no financial tie disclosure. All categories that applied were coded.

### Statistical Analysis

We report frequencies of the different characteristics of each article. For characteristics with sufficient variability, we determine whether particular characteristics were associated with favorable results or conclusions. We used univariate, exact logistic regression to identify associations between explanatory variables and favorable results and conclusions. Odds ratios (ORs), which report the ratio between the odds of having an event or outcome and the odds of not having an event or outcome, were estimated. Our intention was to control for potential effects of multiple variables simultaneously by using multivariate logistic regression for all variables with p<0.05 in the univariate models. However, based on the few univariate significant associations identified and small sample size, we did not conduct this analysis. Data was analyzed with SAS software (version 9.1, SAS Institute, Cary, NC, USA).

## Results

### Characteristics of included studies

**Figure 1:**
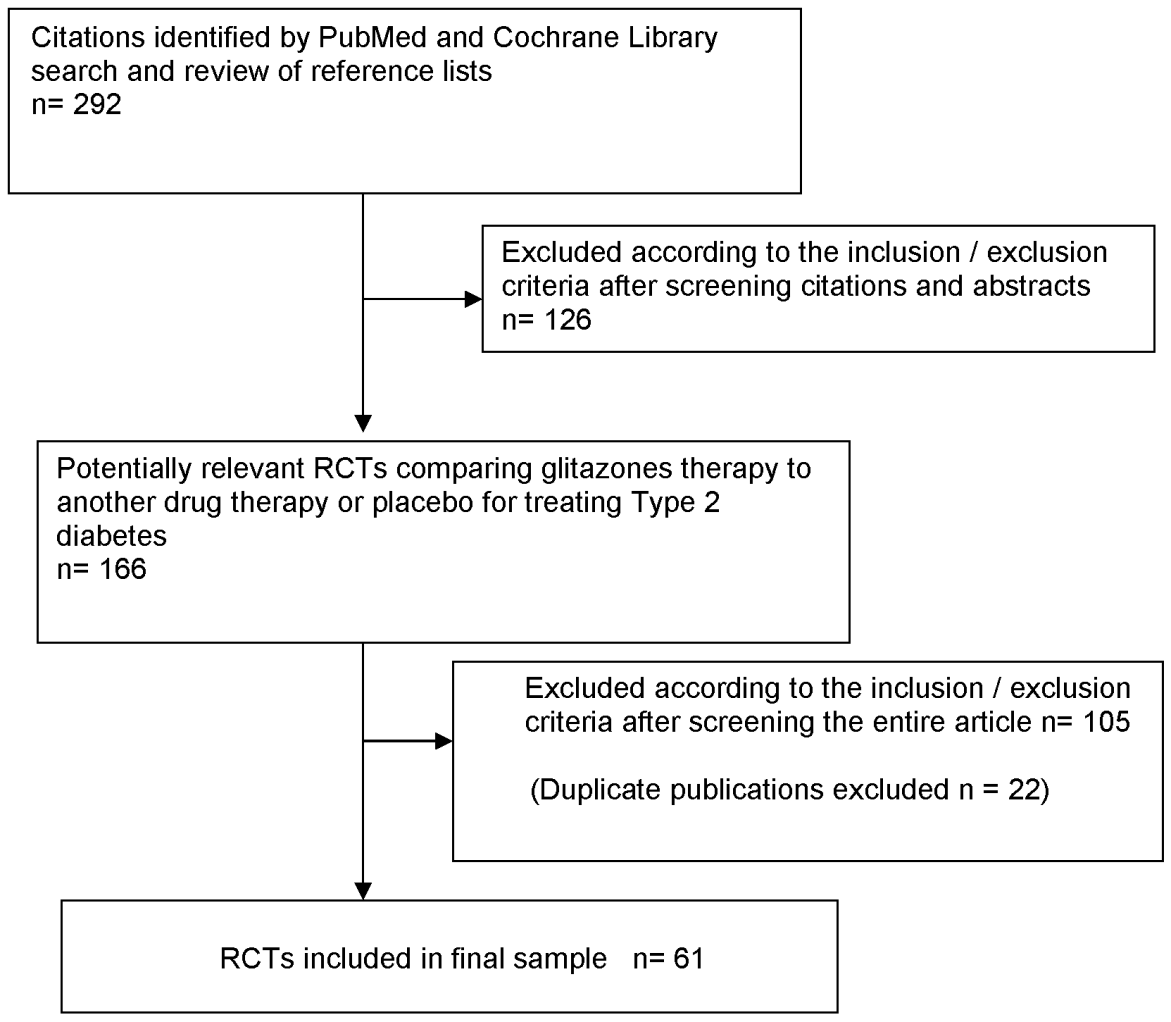
Flowchart for Manuscript selection/ and inclusion.

Our final sample was comprised of 61 published RCTs (Figure 1). The characteristics of included articles by funding source are shown in Table 1. All trials reported glycemic control results and 49 (80%) of trials reported lipid level results. Little variability was found in peer review status or appropriate medication dosing. Only 33 (54%) of the trials were drug-drug comparison trials, while the rest were placebo-controlled trials. Of the 33 drug-drug comparisons, 2 also had placebo arms. We did not code these placebo arms and they are not included in our results. Six of the 61 trials (9.8%) were non-inferiority designs. Seventy-nine percent of trials had conclusions that favored the glitazone or “test” drug, while 69% reported favorable glycemic control results. Of the 13 trials with conclusions that did not favor the test (glitazone) drug, 4 had glycemic control results favoring the glitazone. Moreover, there were 13 trials where the results reported for glycemic control were inconsistent with the conclusions of the papers [29]–[41]. Specifically, in 9 trials, favorable conclusions were reported yet glycemic control results were either similar or unfavorable to the comparator [29]–[37], though notably two trials were focused on lipid effects or inflammatory markers [36], [37]. In 4 trials, the conclusions stated that the comparison drugs were equivalent, but the results reported favorable glycemic control for the glitazone [38]–[41], though one of these trials was focused on lipid effects and inflammatory markers [41]. Funding source was not disclosed in 24 (39%) of the trials. Among trials with disclosed funding, 30 trials had some funding from a glitazone manufacturer and 26 of these were funded exclusively by a glitazone manufacturer. Only 6 of the 37 trials, where funding source was disclosed, were funded by comparator drug companies exclusively and only one of these 37 trials declared no drug company funding.

**Table 1:** Characteristics of included articles by Funding Source (n = 61).

Characteristic	Funding Source				
	Total	Test Drug (Glitazone) Company*	Other Drug Company	All Other	No Funding Disclosure
	N = 61	N = 30	N = 6	N = 1	N = 24
	N (%)	N (%)	N (%)	N (%)	N (%)
**Journal Characteristics**					
Peer-reviewed	59 (97)	29 (97)	6 (100)	1(100)	23 (96)
Impact factor N = 56**					
IF, Quartiles**					
Q1: < = 2	14 (25)	7 (50)	0 (0)	1 (7)	6 (43)
Q2: 2.01–2.84	14 (25)	6 (43)	0 (0)	0 (0)	8 (57)
Q3: 2.85–5.75	14 (25)	8 (57)	2 (14)	0 (0)	4 (29)
Q4: >5.75	14 (25)	6 (43)	4 (29)	0 (0)	4 (29)
**Study Characteristics**					
Comparison groups					
Drug-Drug Comparison	33 (54)	17 (57)	5 (83)	0 (0)	11 (46)
Placebo Control Trial	28 (46)	13 (43)	1(17)	100 (1)	13 (54)
Sample size***					
Sample size***, quartile					
Q1: < = 114	16 (26)	8 (50)	1 (6)	0 (0)	7 (44)
Q2: 115–252	15 (25)	6 (40)	3 (20)	1 (7)	5 (33)
Q3: 253–408	15 (25)	8 (53)	1 (7)	0 (0)	6 (40)
Q4: >408	15 (25)	8 (53)	1 (7)	0 (0)	6 (40)
**Study design characteristics aimed at reducing bias**					
Adequate randomization & concealment of allocation	15 (25)	9 (30)	1 (17)	0 (0)	5 (21)
Adequate blinding	36 (59)	18 (60)	2 (33)	0 (0)	15 (63)
Adequate intention to treat analysis	48 (79)	25 (83)	5 (83)	0 (0)	18 (75)
Adequate follow-up	48 (79)	23 (77)	5 (83)	1(100)	19 (79)
**Appropriate medication dosing**					
Test drug (glitazone) n = 61	60 (98)	30 (100)	5 (83)	1 (100)	24 (100)
Comparator drug n = 33	30 (90)	16 (94)	5 (100)	–	9 (82)
		n = 17	n = 5	n = 0	n = 11
**Results**					
Glycemic Control n = 61					
Favorable to test drug (glitazone)	42 (69)	22 (73)	2 (33)	1 (100)	17 (71)
Lipid Level n = 49					
Favorable to test drug	25 (51)	16 (70)	0 (0)	0 (0)	9 (45)
		n = 23	n = 5	n = 1	n = 20
**Conclusions** n = 61					
Favors test drug (glitazone)	48 (79)	26 (87)	1 (17)	1 (100)	20 (83)
**Author characteristics**					
Corresponding author institutional affiliation					
Test drug (glitazone)	16 (26)	11 (37)	0 (0)	0 (0)	5 (21)
Any other drug company	1 (2)	0 (0)	0 (0)	0 (0)	1 (4)
Academic/Hospital	43 (70)	19 (63)	6 (100)	1 (100)	17 (71)
All other/can't determine	1 (2)	0 (0)	0 (0)	0 (0)	1 (4)

* Includes articles that were sponsored by glitazone company, another drug company and other non -drug company funding (n = 2) and by glitazone company and non-drug-company (n = 2).

** Data from 5 articles were excluded because they were published in journals that had no impact factor. Of the N = 56 trials reported in journals with impact factors, median value N = 2.84, mean value = 4.63, range (0.34–44.02) and standard deviation σ = 6.06.

*** Sample size characteristics, median value N = 252, mean value = 390, range (20–4360) and standard deviation σ = 590.4.

### Association of trial characteristics with results or conclusions that favor glitazones

Results of the univariate exact logistic regression analysis used to identify associations between favorable results or conclusions and explanatory variables are given in Tables 2 and 3 respectively. Studies that report favorable glycemic control results were about 5 times more likely to be adequately blinded than not and four times more likely to have a corresponding author with financial ties to the glitazone manufacturer. Additionally, studies that reported lipid level results favorable to the glitazone were about four times more likely to be funded by a glitazone manufacturer than another sponsor (Table 2). Studies that reported conclusions favoring the glitazone were 0.03 times as likely to be funded by a comparator drug company and 0.8 times as likely to be published in journals with higher (better) impact factors (Table 3).

**Table 2:** Association between characteristics of articles and statistically significant glycemic control and lipid level results that favor the test drug: Univariate exact logistic regression.

	Glycemic Control Results	Odds Ratio (95% CI)	P value	Lipid Level Results	Odds Ratio (95% CI)	P value
	**that favor test drug (glitazone)**			**that favor test drug (glitazone)**		
	**Favorable N/Total N (%)**			**Favorable N/Total N (%)**		
**Journal Characteristics**						
**Journal Impact factor**						
Range 0.34–44.02	40/56 (71)	1.010 (0.912, 1.130)	0.8902	23/56 (41)	0.924 (0.742, 1.138)	0.4789
Mean Value = 4.63						
Std deviation = 6.06						
Median Value = 2.84						
**Study characteristics**						
**Comparison Groups**						
Placebo Controlled trial	27/28 (96)	1.00		11/25 ( 44)	1.00	
Drug–drug Comparative trial	15/33 (46)	0.031 (0 , 0.245)	0	14/24 (58)	1.782 (0.499, 6.425)	0.4736
**Sample size**						
Range 20–4360	42/61	1.002 (1.000, 1.005)	0.0394	25/49	0.999 (0.997, 1.001)	0.3720
Mean Value = 390						
Std deviation = 590.4						
Median Value = 252						
**Study design characteristics aimed at reducing bias**						
**Randomization/Concealment of allocation**						
Adequate	11/15 (73)	1.331 (0.320, 6.669)	0.9292	5/10 (50)	0.950 (0.186, 4.868)	1.000
**Blinding**						
Adequate	30/36 (83)	5.417 (1.459, 21.188)	0.0081	11/28 (39)	0.324 (0.083, 1.216)	0.1066
**ITT Analysis**						
Adequate	36/48 (75)	4.200 (0.922, 19.750)	0.0664	18/38 (47)	0.514 (0.095, 2.464)	0.5453
**Follow-up**						
Adequate	31/49 (63)	0.157 (0.003, 1.274)	0.1061	20/40 (50)	0.800 (0.138, 4.358)	1.0000
**Appropriate Medication Dosing**						
Test drug – Adequate	42/60 (70)	Infinite (0.057, Infinite)	0.6230	25/48 (52)	Infinite (0.027, Infinite)	0.9796
Comparator drug - Adequate	14/30 (47)	1.750 (0.081, 110.507)	1.0000	12/21 (57)	0.667 (0.010, 14.997)	1.0000
**Author characteristics**						
**Corresponding Author Institutional Affiliation**						
Test drug company	16/16 (100)	Infinite (0.410, Infinite)	0.1176	8/13 (62)	Infinite (0.034, Infinite)	0.8571
Any other industry	0/1 (0)	–	–	0/1 (0)		
Academic/Hospital	26/43 (61)	Infinite (0.037, Infinite)	0.8182	17/35 (49)	Infinite (0.023, Infinite)	1.0000
All Other	0/1 (0)	1.000		0/0 (0)		
**Funding information**						
**Funding source**						
Test drug company/any other industry/other drug companies	0/2 (0)	0 (0, 1.656)	0.1111	2/2 (100)	Infinite (0.092, Infinite)	0.8667
Test drug company employee author/no formal disclosure	1/2 (50)	0.238 (0.003, 22.399)	0.7778	2/2 (100)	Infinite (0.092, Infinite)	0.8667
Any other drug company	2/6 (33)	0.119 (0.009, 1.190)	0.0769	0/5 (0)	0 (0, 0.886)	0.0373
No drug company	1/1 (100)	Infinite (0.006, Infinite)	1.0000	0/1 (0)	0 (0, 26.000)	0.8000
No funding disclosure	17/24 (71)	0.578 (0.122, 2.590)	0.6238	9/20 (45)	0.477 (0.109, 2.043)	0.4153
**Test drug funding**						
Test drug funding	22/30 (73)	1.512 (0.445, 5.269)	0.6416	16/23 (70)	4.317 (1.126, 17.134)	0.0300
**Corresponding Author Financial Ties**						
Test drug company ties/any other industry ties	2/5 (40)	0.133 (0.002, 3.502)	0.3939	2/3 (67)	2.000 (0.061, 156.747)	1.0000
Test drug company employee/no formal disclosure of ties	15/15 (100)	Infinite (0.064, Infinite)	0.5714	8/12 (67)	2.000 (0.173, 22.393)	0.8552
Any other industry ties	2/2 (100)	Infinite (0.009, Infinite)	1.0000	1/2 (50)	1.000 (0.010, 104.374)	1.0000
No disclosure of ties	18/32 (56)	0.257 (0.005, 2.776)	0.4388	10/25 (40)	0.667 (0.074, 6.103)	0.9987
**Corresponding Author Test Drug Financial Ties**						
Test drug financial ties	22/26 (85)	4.125 (1.048, 19.525)	0.0409	13/21 (62)	2.167 (0.592, 8.099)	0.3025
**First Author Financial Ties**						
Test drug company ties/any other industry ties	2/5 (40)	0 (0, 2.027)	0.1667	2/3 (67)	1.333 (0.036, 117.498)	1.0000
Test drug company employee/no formal disclosure of ties	5/5 (100)	1.00		3/5 (60)	1.000 (0.042, 23.663)	1.0000
Any other industry ties	2/2 (100)	1.00		1/2 (50)	0.667 (0.006, 78.249)	1.0000
No disclosure of ties	28/44 (64)	0 (0, 2.188)	0.2489	16/34 (47)	0.593 (0.045, 5.955)	0.9492
**First Author Test Drug Financial Ties**						
Test drug financial ties	12/15 (80)	2.133 ((0.469, 13.331)	0.4588	8/13 (62)	1.788 (0.414, 8.297)	0.5762

**Table 3:** Association between characteristics of articles and statistically significant author's conclusions that favor the test drug: Univariate exact logistic regression.

	Conclusions **that favor test drug (glitazone)**	Odds Ratio (95% CI)	P value
	**Favorable N/Total N (%)**		
**Journal characteristics**			
**Journal Impact factor**			
Range 0.34–44.02	45/56 (80)	0.794 (0.619, 0.967)	0.0109
Mean Value = 4.63			
Std deviation = 6.06			
Median Value = 2.84			
**Study characteristics**			
**Comparison Groups**			
Placebo Controlled trial	25/28 (89)	1.00	
Drug–drug Comparative trial	23/33 (70)	0.276 (0.044, 1.277)	0.1178
**Sample size**			
Range 20–4360	48/61 (79)	1.000 (0.999, 1.001)	0.4099
Mean Value = 390			
Std deviation = 590.4			
Median Value = 252			
**Study design characteristics aimed at reducing bias**			
**Randomization/Concealment of allocation**			
Adequate	13/15 (87)	2.043 (0.362, 21.213)	0.6354
**Blinding**			
Adequate	31/36 (86)	2.918 (0.698, 13.026)	0.1689
**ITT Analysis**			
Adequate	39/48 (81)	1.445 (0.208, 7.501)	0.8959
**Follow-up**			
Adequate	38/49 (78)	0.691 (0.065, 4.051)	1.0000
**Appropriate Medication Dosing**			
Test drug – Adequate	48/60 (80)	Infinite (0.095, Infinite)	0.4262
Comparator drug - Adequate	20/30 (67)	0 (0, 5.654)	0.6492
**Author characteristics**			
**Corresponding Author Institutional Affiliation**			
Test drug company	15/16 (94)	Infinite (0.192, Infinite)	0.2353
Any other industry	0/1 (0)	–	–
Academic/Hospital	33/43 (77)	Infinite (0.077, Infinite)	0.5000
All Other	0/1 (0)	1.000	–
**Funding information**			
**Funding source**			
Test drug company/any other industry/other drug companies	1/2 (50)	0.130 (0.002, 13.627)	0.5397
Test drug company employee author/no formal disclosure	2/2 (100)	Infinite (0.020, Infinite)	1.0000
Any other drug company	1/6 ((17)	0.026 (0, 0.400)	0.0030
No drug company	1/1 (100)	Infinite (0.003, Infinite)	1.0000
No funding disclosure	20/24 (83)	0.652 (0.086, 4.407)	0.9068
**Test drug funding**			
Test drug funding	26/30 (87)	2.659 (0.623, 13.280)	0.2357
**Corresponding Author Financial Ties**			
Test drug company ties/any other industry ties	2/5 (40)	0 (0, 1.644)	0.1212
Test drug company employee/no formal disclosure of ties	15/15 (100)	1.000	–
Any other industry ties	1/2 (50)	0 (0, 13.000)	0.5000
No disclosure of ties	23/32 (72)	0 (0, 2.705)	0.3441
**Corresponding Author Test Drug Financial Ties**			
Test drug financial ties	23/26 (89)	3.067 (0.664, 19.156)	0.1937
**First Author Financial Ties**			
Test drug company ties/any other industry ties	2/5 (40)	0 (0, 2.027)	0.1667
Test drug company employee/no formal disclosure of ties	5/5 (100)	1.000	–
Any other industry ties	1/2 (50)	0 (0, 15.600)	0.5714
No disclosure of ties	35/44 (80)	0 (0, 5.075)	0.6901
**First Author Test Drug Financial Ties**			
Test drug financial ties	12/15 (80)	1.111 (0.230, 7.302)	1.0000

## Discussion

We examined the association between study design characteristics and the results and conclusions of randomized controlled trials of the currently marketed thiazolidinediones, pioglitazone and rosiglitazone, versus either placebo or comparator drug. Our hypothesis was that results and conclusions of published trials would be more likely to favor the study sponsor’s drug or that of the company with which an author had financial ties, and that other design features would also be associated with results or conclusions that favor the study sponsor’s drug. Our study adds more support to the body of literature demonstrating that pharmaceutical industry sponsored studies comparing drug and placebo are more likely to favor the drug [5]–[8] and that the sponsor’s drug in a drug-drug comparison is more likely to be favored [2], [13]. Further, our finding suggests that favorable results and conclusions are associated with the financial ties the corresponding author has with the sponsor of the study. We also found that less favorable results and conclusions were obtained when the study sponsor was a competitor. This may explain why well-designed comparisons of thiazolidinediones (glitazones) versus other type 2 diabetes drugs sometimes have contradictory results when executed by different investigators with financial ties to different pharmaceutical sponsors.

Several plausible explanations exist for our finding of the strong association between favorable outcomes and corresponding author financial ties or funding source for the study. Publication bias or the fact that statistically significant results are published more often than non-statistically significant results, may explain this association as we only examined published studies [4]. Moreover, corresponding authors with financial ties to a sponsor may be less likely to publish results that are unfavorable to the sponsor. We also found that adequate blinding and larger sample size was associated with favorable glycemic control results. Thus, these two characteristics of good trial design may be related to the industry sponsorship. Recent research has shown that specific study design characteristics are not reliably associated with treatment effect sizes across different studies and medical areas [42].

Journal characteristics may influence the results and conclusions of articles as the quality of reporting can vary with the journal. Articles published in peer-reviewed journals have superior quality compared to articles published in non-peer reviewed journals [7]. In our sample there was no variability in peer review status. However, we did find a significant association between favorable conclusions and journal impact factor. Articles published in higher impact journals were less likely to reach favorable conclusions than articles published in journals with lower impact factors.

Our study had several limitations. Although we conducted a comprehensive search, we identified only 61 trials for inclusion. Our intention was to control for potential effects of multiple variables simultaneously by using multivariate logistic regression for all variables with p<0.05 in the univariate models. However, based on the few univariate significant associations identified and our small sample size, it was inappropriate to conduct this analysis. Furthermore, due to the few significant associations detected, we did not correct p-values in the univariate analysis for multiple statistical tests. Second, we searched PubMed and the Cochrane Library, but not other databases, such as Embase, so we may have missed some published manuscripts relevant to our study. Third, we categorized studies as funded by industry based on each article’s disclosure and a large percentage (39%) of studies in our sample had no disclosed funding sources. This lack of disclosure could underestimate the number of industry sponsored studies and personal ties of investigators [43]. Additionally, our study only included randomized controlled trials and no observational studies, and did not include any head-to-head comparisons of one glitazone to another. Consequently, we cannot assess whether or not one glitazone is favored over the other.

We found that the published trials are dominated by industry sponsored studies. There is increasing concern that funding source influences outcomes and conclusions of medical research. Over the last few decades the amount of industry funded medical research has increased dramatically and consequently could affect the balance of published trials towards studies that favor new drugs over older generic drugs [44], [45]. More diversity in glitazone trial funding seems warranted. Moreover, we found a very large percentage of placebo-controlled trials which are not appropriate comparisons for determining the value of a glitazone as an add-on therapy for treating type 2 diabetes. We also found few head-to-head comparisons of drug regimens containing glitazones versus drug regimens containing insulin, which leaves clinicians unable to evaluate the effectiveness of one combination regimen over another.
